# The Structure, Function, and Mechanisms of Action of Enterovirus Non-structural Protein 2C

**DOI:** 10.3389/fmicb.2020.615965

**Published:** 2020-12-14

**Authors:** Shao-Hua Wang, Kuan Wang, Ke Zhao, Shu-Cheng Hua, Juan Du

**Affiliations:** ^1^Institute of Virology and AIDS Research, The First Hospital of Jilin University, Changchun, China; ^2^Department of Neurotrauma, The First Hospital of Jilin University, Changchun, China; ^3^Department of Internal Medicine, The First Hospital of Jilin University, Changchun, China; ^4^Key Laboratory of Organ Regeneration and Transplantation of the Ministry of Education, The First Hospital of Jilin University, Changchun, China

**Keywords:** enterovirus, 2C protein, structure, function, host immune response, type I IFNs, anti-viral drug

## Abstract

Enteroviruses are a group of RNA viruses belonging to the family *Picornaviridae*. They include human enterovirus groups A, B, C, and D as well as non-human enteroviruses. Enterovirus infections can lead to hand, foot, and mouth disease and herpangina, whose clinical manifestations are often mild, although some strains can result in severe neurological complications such as encephalitis, myocarditis, meningitis, and poliomyelitis. To date, research on enterovirus non-structural proteins has mainly focused on the 2A and 3C proteases and 3D polymerase. However, another non-structural protein, 2C, is the most highly conserved protein, and plays a vital role in the enterovirus life cycle. There are relatively few studies on this protein. Previous studies have demonstrated that enterovirus 2C is involved in virus uncoating, host cell membrane rearrangements, RNA replication, encapsidation, morphogenesis, ATPase, helicase, and chaperoning activities. Despite ongoing research, little is known about the pathogenesis of enterovirus 2C proteins in viral replication or in the host innate immune system. In this review, we discuss and summarize the current understanding of the structure, function, and mechanism of the enterovirus 2C proteins, focusing on the key mutations and motifs involved in viral infection, replication, and immune regulation. We also focus on recent progress in research into the role of 2C proteins in regulating the pattern recognition receptors and type I interferon signaling pathway to facilitate viral replication. Given these functions and mechanisms, the potential application of the 2C proteins as a target for anti-viral drug development is also discussed. Future studies will focus on the determination of more crystal structures of enterovirus 2C proteins, which might provide more potential targets for anti-viral drug development against enterovirus infections.

## Introduction

The *Enterovirus* (EV) genus consists of a large number of RNA viruses belonging to the family *Picornaviridae*, including human enterovirus groups A, B, C, and D as well as non-human enteroviruses (Zoll et al., 2009). Of these pathogens, enterovirus A71 (EV-A71) and coxsackievirus A16 (CV-A16) are the most common causative pathogens of hand, foot, and mouth disease (HFMD), which affect millions of people each year, especially infants and children under of 5 years age, in the Asian and Pacific regions (Wang et al., 2018). Although usually self-limiting, HFMD can lead to severe complications such as aseptic meningitis, acute flaccid paralysis, and neurological respiratory syndrome or fatal respiratory disease (Chang et al., 1998; Zell et al., 2017). However, other non-EV-A71 and non-CV-A16 human EV-A group pathogens, such as CV-A6, CV-A10, and CV-A4, are predominant co-circulated serotypes which have been causing HFMD in China since 2013 (He et al., 2013; Li et al., 2018a; Ji et al., 2019; Xie et al., 2020).

**Figure 1:**
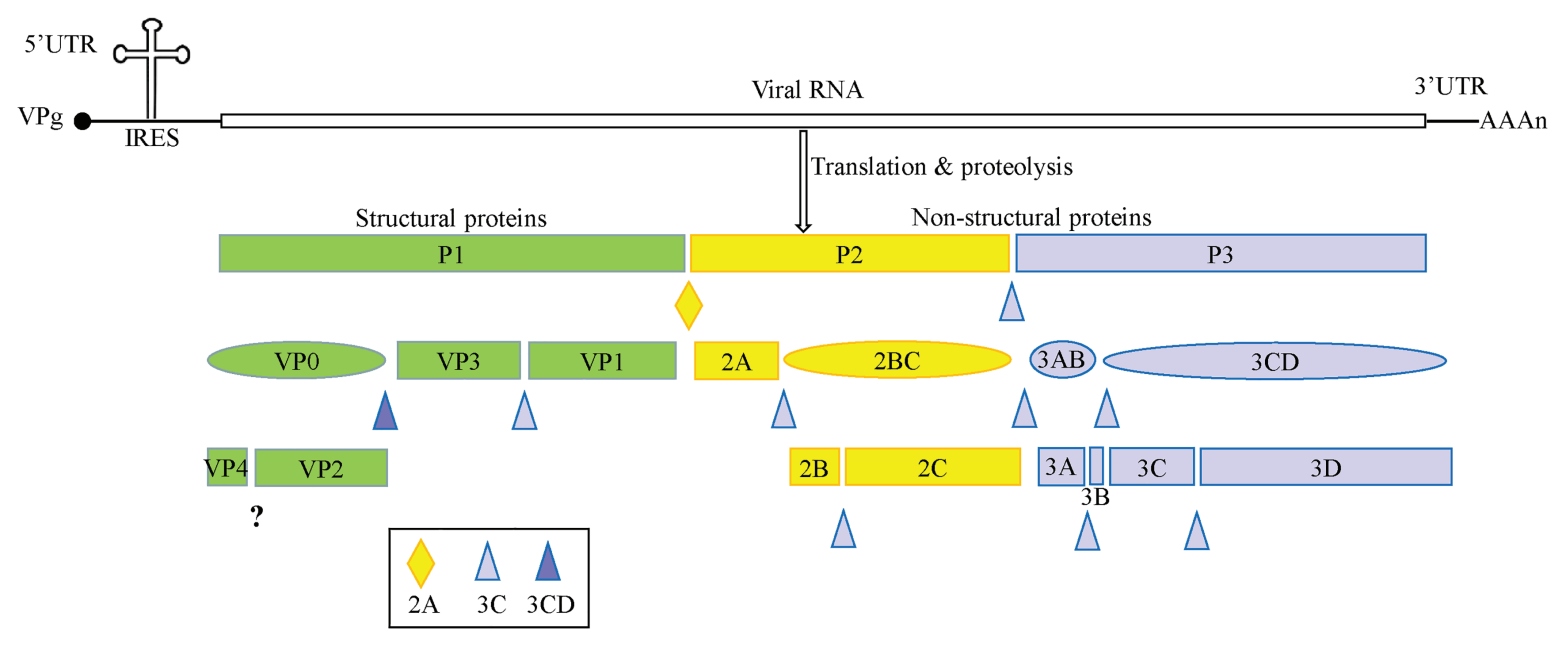
A schematic representation of enterovirus A71 (EV-A71) genome and proteolytic processing of the polyprotein. The polyprotein was cleaved into four viral proteins, VP1–VP4, and seven non-structural proteins including 2A–2C and 3A–3D.

Enteroviruses are non-enveloped, spherical viruses with a diameter ranging from 28 to 30 nm, and single positive-stranded RNA. The genome of these enteroviruses is approximately 7.5–8.0 kb in length and contains one open reading frame (ORF), flanked by a highly structured 5'-untranslated region (5' UTR) and a 3' UTR with a poly(A) tail. The 5' UTR is composed of an RNA cloverleaf structure followed by an internal ribosomal entry site (IRES). The IRES is a highly structured RNA that directly recruits ribosomes for viral protein translation in a cap-independent manner (Fitzgerald and Semler, 2009). As shown in Figure 1, the genome is initially translated into a single large polyprotein of approximately 2,200 amino acid residues. This polyprotein is proteolyzed into P1, P2, and P3 precursor proteins, and is further cleaved co- and post-translationally by viral 2A, 3C, and 3CD proteases (Cameron et al., 2010; Lyoo et al., 2017). The P1 precursor protein is cleaved into the capsid proteins VP3, VP1, and VP0. VP0 is then further divided into VP4 and VP2. The P2 precursor protein is processed to form the viral protease 2A and the 2BC polyprotein, and the 2BC polyprotein is further cleaved into two non-structural proteins, 2B and 2C. The P3 precursor protein is initially proteolyzed into 3AB and 3CD, and then further proteolyzed to form proteins 3A, 3B, 3C, and 3D (McMinn, 2002).

Most published studies have focused on enterovirus structural proteins or the non-structural 2A and 3C proteases, and 3D polymerase, whereas the importance of the non-structural protein 2C has been relatively neglected. In this article, we summarize the structure, function, and mechanism of regulation of the host innate immune system and anti-viral drugs of the enterovirus 2C protein.

## Common Function of Enterovirus 2C Proteins

Enterovirus 2C protein is the most conserved and complex non-structural protein, but its functions are not well understood (Norder et al., 2011). Numerous biological functions of the 2C protein have been reported as part of the virus life cycle (Table 1), including virus uncoating (Li and Baltimore, 1990), host cell membrane rearrangements (Cho et al., 1994; Aldabe and Carrasco, 1995; Teterina et al., 1997; Suhy et al., 2000), RNA binding (Rodriguez and Carrasco, 1995; Banerjee et al., 1997, 2001; Banerjee and Dasgupta, 2001), RNA replication (Li and Baltimore, 1988; Rieder et al., 2000; Paul et al., 2003; Teterina et al., 2006; Tang et al., 2007), encapsidation and morphogenesis (Vance et al., 1997; Verlinden et al., 2000; Liu et al., 2010; Wang et al., 2012a, 2014), and ATPase activity (Rodriguez and Carrasco, 1993; Mirzayan and Wimmer, 1994).

**Table 1:** The function of enterovirus non-structural 2C proteins.

Protein	Genus	Species	Functions	References
2C	*Enterovirus*	PV	Virus uncoating	Li and Baltimore, 1990
2C	*Enterovirus*	PV	Membrane rearrangements	Cho et al., 1994; Aldabe and Carrasco, 1995; Teterina et al., 1997; Suhy et al., 2000
2C	*Enterovirus*	PV; HAV, RV-14	RNA binding	Rodriguez and Carrasco, 1995; Banerjee et al., 1997, 2001; Banerjee and Dasgupta, 2001
2C	*Enterovirus*	PV; EV-A71	RNA replication	Li and Baltimore, 1988; Rieder et al., 2000; Paul et al., 2003; Teterina et al., 2006; Tang et al., 2007
2C	*Enterovirus*	PV	Encapsidation	Vance et al., 1997; Verlinden et al., 2000
2C	*Enterovirus*	PV	Morphogenesis	Liu et al., 2010; Wang et al., 2012a, 2014
2C	*Enterovirus*	PV	ATPase activity	Rodriguez and Carrasco, 1993; Mirzayan and Wimmer, 1994
2C	*Enterovirus*	PV; EV-A71, CV-A16	Helicase activity	Gorbalenya and Koonin, 1989; Rodriguez and Carrasco, 1993; Pfister and Wimmer, 1999; Xia et al., 2015
2C	*Enterovirus*	EV-A71, CV-A16	RNA chaperoning activities	Xia et al., 2015

The 2C protein was predicted to be an SF3 helicase, based on its AAA+ ATPase activity and conserved SF3 motifs (Gorbalenya and Koonin, 1989; Rodriguez and Carrasco, 1993; Pfister and Wimmer, 1999). In 2015, it was first demonstrated that the 2C protein of EV-A71 and CV-A16 possesses ATP-dependent RNA helicase and ATP-independent chaperoning activities, which are critical for viral RNA replication (Xia et al., 2015). These results indicate that the RNA helicase and RNA chaperoning activities, two different RNA remodeling activities, can be integrated in the 2C protein, suggesting a vital role for the 2C protein in the remodeling of proteins by viral RNA (Xia et al., 2015).

## The Relationship Between Structure and Function of 2C Proteins in the Enterovirus Life Cycle

**Figure 2:**
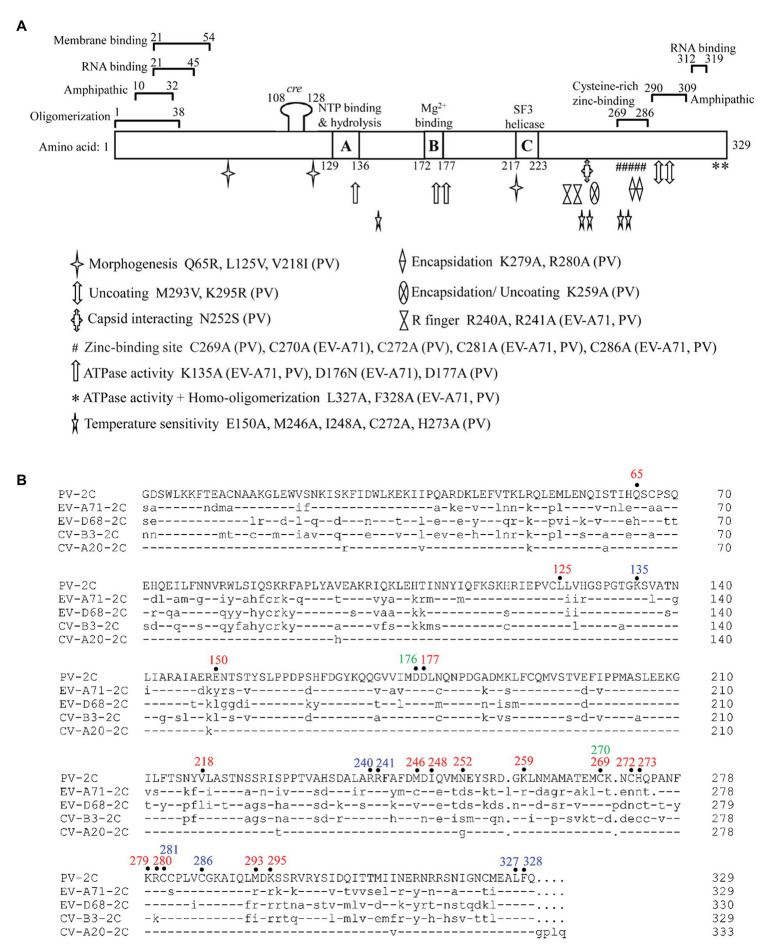
Functional motifs and sequence alignment of enterovirus 2C protein. **(A)** Functional motifs in 2C^ATPase^ protein are shown in detail, including membrane binding-, RNA binding-, zinc binding-, oligomerization-, and amphipathic-motifs. The locations of the known mutations corresponding to morphogenesis, encapsidation, uncoating, capsid interacting, ATPase activity, homo-oligomerization, and temperature sensitivity are shown in different symbols. The precise positions of R finger and zinc-binding sites are indicated according to the crystal structural of EV-A71 2C protein. Amino acid positions in each motif are numbered and illustrated, and the exact species of each mutation is shown behind the amino acid position. **(B)** Sequence alignment of enterovirus 2C protein. The amino acid positions mentioned in **(A)** are highlighted on top of the sequences in different colors, with PV 2C in red, EV-A71 2C in green, and positions shared by both in blue. Dashes stand for amino acid residues identical to those of PV 2C protein.

The 2C protein typically has 330 amino acid residues. It contains an N-terminal membrane-binding domain, a central ATPase domain, a cysteine-rich domain, and a C-terminal helical domain (Figure 2A; Banerjee et al., 2004). The ATPase domain of 2C exhibits the structural characteristics of the SF3 helicases of the AAA+ ATPase superfamily, which consists of Walker A and Walker B motifs, and motif C (Figure 2B; Singleton et al., 2007). Recently, the crystal structure of a soluble part (116–329 aa) of EV-A71 2C helicase was reported by Guan et al. (2017), the first high-resolution 2C structure in the *Picornaviridae* family. EV-A71 2C has an unusual zinc finger with three cysteine ligands. However, unlike other ATPases, the C-terminus of EV-A71 2C forms an amphipathic helix that mediates self-oligomerization through a specific interaction between 2C-2C, and self-oligomerization is fundamental to 2C ATPase activity and EV-A71 virus replication.

Poliovirus (PV) 2C protein is the most intensively studied 2C protein in the *Picornaviridae* family. Recently, Guan et al. (2018) reported a high-resolution structure of part of the PV 2C protein (116–329 aa). Their results indicated that the self-oligomerization mediated by the C-terminal helix of the PV 2C protein also occurred via a specific interaction between 2C-2C, like that of EV-A71 2C. This interaction is vital to the protein’s ATPase activity, and is a common feature in enterovirus 2C proteins. PV 2C and EV-A71 2C possess almost identical geometry, and catalytic residues of the ATPase active site, which forms between these 2C subunits, and should have similar functions (Mirzayan and Wimmer, 1994; Pfister and Wimmer, 1999). However, the part of the protein which is most structurally different between PV 2C and EV-A71 2C is the zinc finger. PV 2C has four potential zinc coordination sites (PCS1–4) in the cysteine-rich motif, suggesting a canonical CCCC type zinc finger, while EV-A71 2C and many other enterovirus 2C proteins have only a CCC type zinc finger, which lacks the PCS2 cysteine residue (Figure 2B). It has been reported that the PCS2 of PV 2C is associated with temperature-sensitive phenotypes and encapsidation defects (Klein et al., 2000; Wang et al., 2014), but the zinc finger of EV-A71 2C, to which the PCS2 has been added by mutation, failed to improve its infectivity. Further studies indicated that PCS2 and PCS4 might interact with other proteins during encapsidation, while PCS1 and PCS3 are essential for maintaining the folding of the zinc finger and the entire hexamer (Guan et al., 2018). Therefore, the sequence and structure distinction of the 2C protein may be the basis for the specificity of the enterovirus 2C protein and may determine the processes in which it may be involved.

Numerous residues, drug-resistant sites, and functional motifs have been identified in the enterovirus non-structural 2C proteins, which are critical to differences in the corresponding 2C function (Figure 2A). The ATPase and helicase activities of the 2C protein are mainly affected by mutations in the Walker A motif (positions 129–136; Wang et al., 2014), Walker B motif (positions 172–177; Wang et al., 2014), Motif C (positions 217–223; Xia et al., 2015), and the R finger (R240 and R241; Guan et al., 2017). The residues buried in the hydrophobic core of the 2C protein were found to be essential for overall folding (Guan et al., 2018). It has been reported that mutations of the 2C protein at positions Q65, L125, and V218 are important to encapsidation and morphogenesis (Vance et al., 1997; Wang et al., 2014; Asare et al., 2016), while mutations at V218, M246, and I248 accounted for temperature sensitivity (Li and Baltimore, 1988; Dove and Racaniello, 1997). The mutations at L327 and F328, which are both located in the pocket-binding domain (PBD), can abolish the ATPase activity and homo-oligomerization of both PV 2C^ATPase^ and EV-A71 2C helicases, and could suppress EV-A71 infection, indicating their essential role in the activity of 2C (Guan et al., 2017, 2018). Residues between 21–45 and 312–319 in the PV 2C protein are critical for RNA binding (Tolskaya et al., 1994), while residues 21–54 are important for membrane binding (Echeverri and Dasgupta, 1995), and positions 269–286 are essential for zinc binding (Klein et al., 2000). In addition, 2C^ATPase^ contains two amphipathic helixes at the N- and C-terminals, which could help anchor the protein to membranes and bind to zinc (Figure 2; Paul et al., 1994; Teterina et al., 1997; Wang et al., 2014).

Liu et al. (2010) found that the interaction site was between residue N252 of PV 2C and E180 of the capsid VP3 protein of CV-A20, using a PV/CV-A20 chimera, indicating the essential role of N252 in encapsidation. The K259A mutant of PV 2C was found to play a vital role in encapsidation and the subsequent uncoating step during the next cycle of infection (Asare et al., 2016). Wang et al. (2012a) identified the K279 and R280 residues, which are located at the C-terminus of the PV 2C protein, as being involved in RNA replication and encapsidation. C270, C281, and C286 of the zinc finger were shown to be essential for correct folding of the EV-A71 2C protein (Guan et al., 2017). Previous studies have shown that both 2C and its precursor 2BC possess ATPase activity and can assist in the formation of an RNA replication complex with which to attach to membranes (Pfister et al., 2000). EV-A71 virus production was completely suppressed by key mutations in K135A and D176N, which are located in the Walker A and B motifs, indicating an important role for the ATPase activity of 2C in virus replication. The ATPase activity of 2C could also be inhibited by mutations at R240 and R241, regardless of whether the amino acid was mutated to A or K, suggesting that the “R finger” may play an essential role in ATP hydrolysis (Guan et al., 2017).

RNA remodelers consist of two different types: RNA helicases and RNA chaperons. It has been reported that these highly structured RNA elements of viruses, especially RNA viruses, utilize RNA helicases or chaperons to ensure proper folding and re-folding (Xia et al., 2015). RNA helicases can unwind RNA duplexes using energy from ATP hydrolysis. However, RNA chaperones are a group of proteins that possess the ability to destabilize RNA duplexes, and could transform them to more stable RNA structures without RNA binding, or using energy from ATP hydrolysis (Musier-Forsyth, 2010; Yang et al., 2015). As with PV 2C^ATPase^, the ATPase and helicase activity of EV-A71 2C could be inhibited by the GK134AA mutation, which abolishes the RNA replication and virus production of EV-A71, suggesting that the RNA remodeling activities introduced by 2C^ATPase^ are essential for enteroviral RNA replication and the life cycle. These RNA remodeling activities are also conserved in CV-A16 2C^ATPase^ (Xia et al., 2015). Further studies revealed that the C-terminal is critical for helicase activity, and the domains that account for RNA binding are required for the RNA chaperoning function of 2C^ATPase^ (Xia et al., 2015).

Internal ribosomal entry site, which is a highly structured element in the RNA genome, has been found within all picornaviruses and plays essential roles in the viral replication and translation process (Shih et al., 2011; Cheng et al., 2013). First, the IRES might need RNA chaperoning activity of 2C^ATPase^ to facilitate RNA strand annealing for proper folding and re-folding during viral replication. In addition, during RNA replication of the viral life cycle, the unwinding of the intermediate dsRNA is essential for efficient recycling of viral RNA template and subsequent progeny viral RNA production. In terms of the RNA replication of enterovirus, it is likely that the unwinding of dsRNA is performed by the RNA helicase activity of the 2C protein, as it was reported that the ATPase activity of EV-A71 2C can facilitate 3D-mediated enteroviral RNA synthesis *in vitro* by promoting the recycling of viral RNA template (Xia et al., 2015). Meanwhile, the defective ATPase and helicase activities of EV-A71 2C could almost abolish RNA replication and virus viability in an infectious clone experiment.

It has been reported that the AAA+ ATPase superfamily usually assembles into hexameric ring structure to perform proper functions (Gai et al., 2004; Enemark and Joshua-Tor, 2006). As EV-A71 2C and PV 2C both belong to this superfamily, they are known to form a hexamer ring, which will facilitate further understanding of 2C functions and provide important sites for the development of 2C inhibitors (Guan et al., 2017, 2018).

Enteroviruses 2C proteins participate in diverse processes and play multiple functions in the viral life cycle, based on the conserved 2C structures. The N-terminal of the 2C protein possesses several essential motifs, which are associated with RNA binding, membrane binding, amphipathic, and oligomerization activities. Future studies will focus on the expression of more soluble full-length 2C proteins and the determination of the crystal structure of enterovirus 2C proteins. Crystallographic data would help us to better understand the relationship between the function and structure of 2C proteins, and to elucidate the detailed mechanism of the role of 2C proteins in virus replication and packaging.

## Enterovirus 2C Protein Binding to Various Host Factors

**Figure 3:**
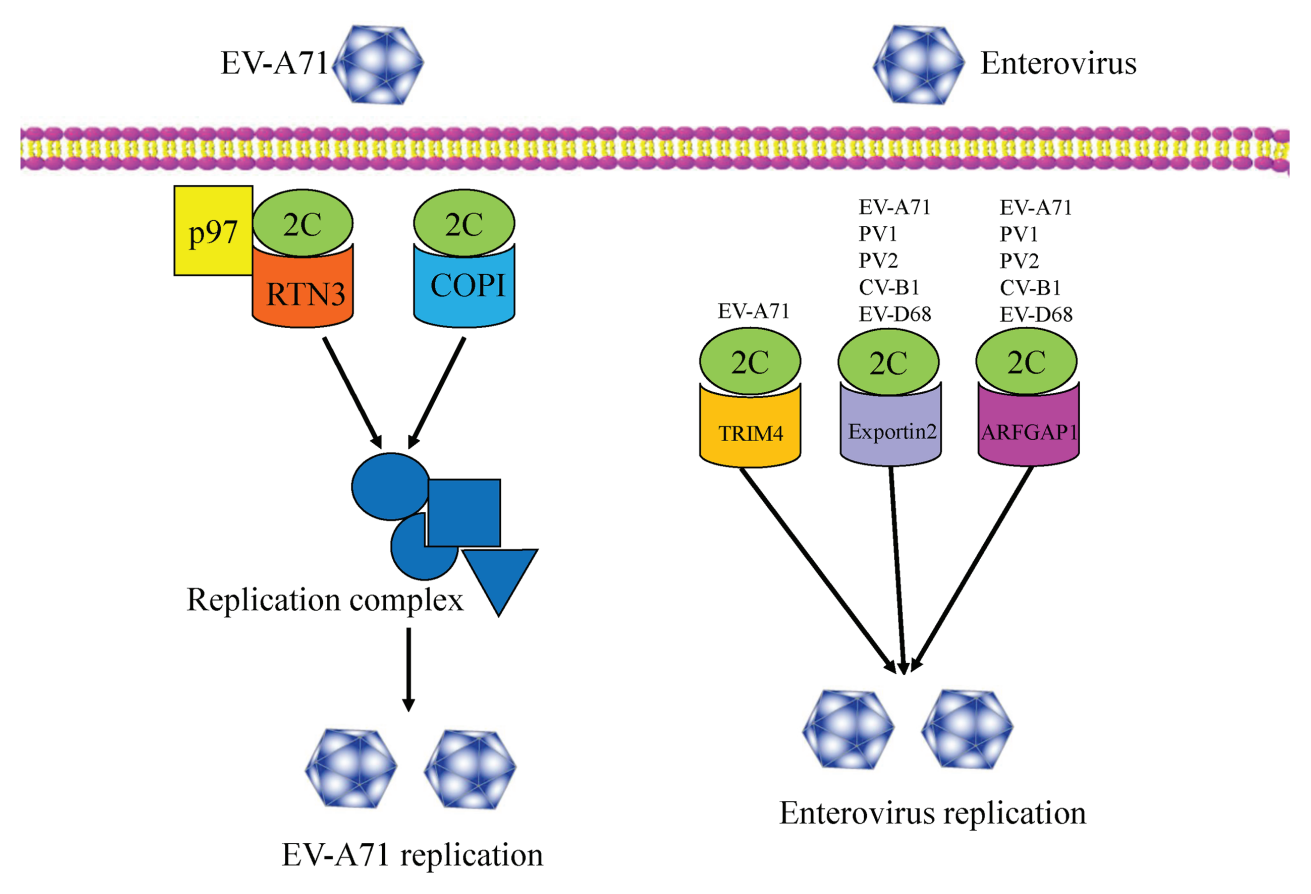
An overview of the functions of enterovirus 2C proteins, which interact with several host factors and regulate viral replication and infection.

Host factors play essential roles in the enterovirus life cycle, from viral entry to lytic release processes (Wu et al., 2016). To date, several host factors associated with the non-structural 2C protein have been reported to regulate viral replication (Figure 3). In 2007, Tang et al. (2007), using a two-hybrid experiment, reported the identification of reticulon 3 (RTN3), a member of the reticulon family of proteins, as a binding partner of the EV-A71 2C protein in regulating the formation of the viral replication complex, a common cellular factor among CV-A16 and PV 2C proteins. These results indicate that the N-terminal of the 2C protein could interact with RTN3. The I25 amino acid residue of EV-A71 2C was found to play a key role in the interaction between 2C and the reticulon homology domain (RHD) of RTN3. A similar function can also be observed in the PV 2C protein, in which the I25K mutation of 2C can regulate viral protein processing and RNA replication (Paul et al., 1994). Specific interactions between the C-terminal RHDs of all four RTN family proteins and EV-A71 2C were also demonstrated (Tang et al., 2007).

In 2016, Wu et al. (2016) used a genome-wide RNAi screen in human RD cells and identified 256 host factors involved in EV-A71 replication. Among these factors, the cell cycle regulators aurora kinase B (AURKB) and cyclin-dependent kinase 6 (CDK6) were shown to be resistance factors restricting EV-A71 infection, with the nuclear egress of CDK6 regulated by EV-A71. However, the endoplasmic reticulum (ER)-associated degradation (ERAD) components, N-glycanase 1 (NGLY1), and valosin-containing protein (VCP) were identified as host-supportive factors that facilitate EV-A71 infection and replication. Further studies revealed the colocalization of NGLY1 and EV-A71 replication complexes at the ER to support EV-A71 replication (Wu et al., 2016). Previous studies have shown that p97 is a host factor for PV (Arita et al., 2012) and is essential for hepatitis C virus (HCV) replication (Yi et al., 2016). Wang et al. reported p97 to be a new host factor, which was an ERAD component and is involved in EV-A71 replication. The mechanism of action involves RTN3 colocalizing with 2C and p97 in EV-A71 infected cells, and thus being redistributed and concentrated in the perinuclear region. RTN3 is, therefore, redistributed from the ER membrane to the viral organelles during EV-A71 infection (Wang et al., 2017). In 2020, Su et al. (2020) revealed that multiple heat shock proteins 70 (HSP70s) were exploited by EV-A71 to participate in all phases of the viral life cycle, therein HSPA9 helped fold and stabilize the 2C protein, and subsequently facilitated the formation of the replication complex.

In addition, coat protein complex I (COPI) and COPII have been reported to be involved in the formation of picornavirus-induced vesicles. Wang et al. (2012b) reported that COPI, but not COPII, is required for EV-A71 replication and production, with the regulation mechanism relying upon the fact that only the 2C protein can interact with the coatomer subunit of COPI. More recently, TRIM4, exportin2, and ARFGAP1 have been identified as novel host factors by a GST pull-down assay using proteomic analysis. These three proteins were validated as 2C binding partners and were demonstrated to be novel host dependency factors for EV-A71 (Li et al., 2019b). In particular, the interactions between 2C-exportin2 and 2C-ARFGAP1 were conserved among other enteroviruses. An understanding of viral-host interactions is important for elucidation of viral pathogenesis, and might provide broad-spectrum anti-viral drug targets for enterovirus infection, so future studies will focus on the discovery of more novel host factors that interact with the 2C proteins. Further studies should also investigate whether these host factors are specifically or generally involved in enteroviruses infections. It is necessary to explore the extent of conservation of these host factors among other enteroviruses and even picornaviruses.

## Effects on the Host Immune Response

The innate immune system is the first line of human defense against foreign and dangerous materials or pathogens, and is linked to the activation and programming of adaptive immune responses (Takeuchi and Akira, 2009). The innate immune system is equipped with pattern recognition receptors (PRRs) to detect invading pathogens (Jin et al., 2018). There are three pathways by which the innate immune system detects and recognizes invading microorganisms (Turvey and Broide, 2010). First, the PRRs recognize foreign pathogens as “microbial non-self” by the detection of pathogen-associated molecular patterns (PAMPs). Second, PRRs can recognize and respond to common metabolic consequences of infection and inflammation with danger-associated molecular patterns (DAMPs; Bianchi, 2007). Lastly, the “missing self” molecules derived from normal healthy cells but not infected cells or microbes can also be recognized by innate immune receptors (Jin et al., 2018). Retinoic acid-inducible gene I (RIG-I)-like receptors (RLRs), Toll-like receptors (TLRs), and NOD-like receptors (NLRs) are the three main PRRs responsible for inducing the production of type I IFNs and inflammatory cytokines, which are important innate immune regulators during viral infections (Akira et al., 2006). To date, the RLRs family has been shown to consist of three members: RIG-I, melanoma differentiation-associated protein 5 (MDA5), and laboratory of genetics and physiology 2 (LGP2; Chen and Ling, 2019). Both RIG-I and MDA5 are intracellular dsRNA sensors. The differences between them are that RIG-I recognizes short double-stranded RNA (dsRNA) or 5′-triphosphate single-stranded RNA (ssRNA) with poly(U/A) motifs during RNA virus infection, whereas MDA5 senses long dsRNA >2 kb or viral RNA lacking 2-O-methylation (Hornung et al., 2006; Kato et al., 2008; Zust et al., 2011; Goubau et al., 2014). RIG-I and MDA5 both contain two N-terminal caspase recruitment domains (CARDs), a central DExD/H box ATPase/helicase domain, and a C-terminal regulatory/repression domain (Li et al., 2016a). After recognizing viral infection, the activated RIG-I and MDA5 release their CARD domain to interact with the same domain of the mitochondrial anti-viral signaling (MAVS, also known as IPS-1, VISA, or CARDIF) protein. The transcription factors IFN regulatory factor 3 (IRF3) and NF-κB are activated by interaction with activated MAVS (Kang et al., 2002; Yoneyama and Fujita, 2008; Fitzgerald et al., 2014). Activated IRF3 and NF-κB subsequently translocate to the nucleus and stimulate the expression of type I IFNs, interferon-stimulated genes (ISG), and inflammatory cytokines (Sato et al., 1998; Yoneyama et al., 1998). Thus, RIG-I and MDA5 play important roles in the activation of the IFN signaling pathway.

**Figure 4:**
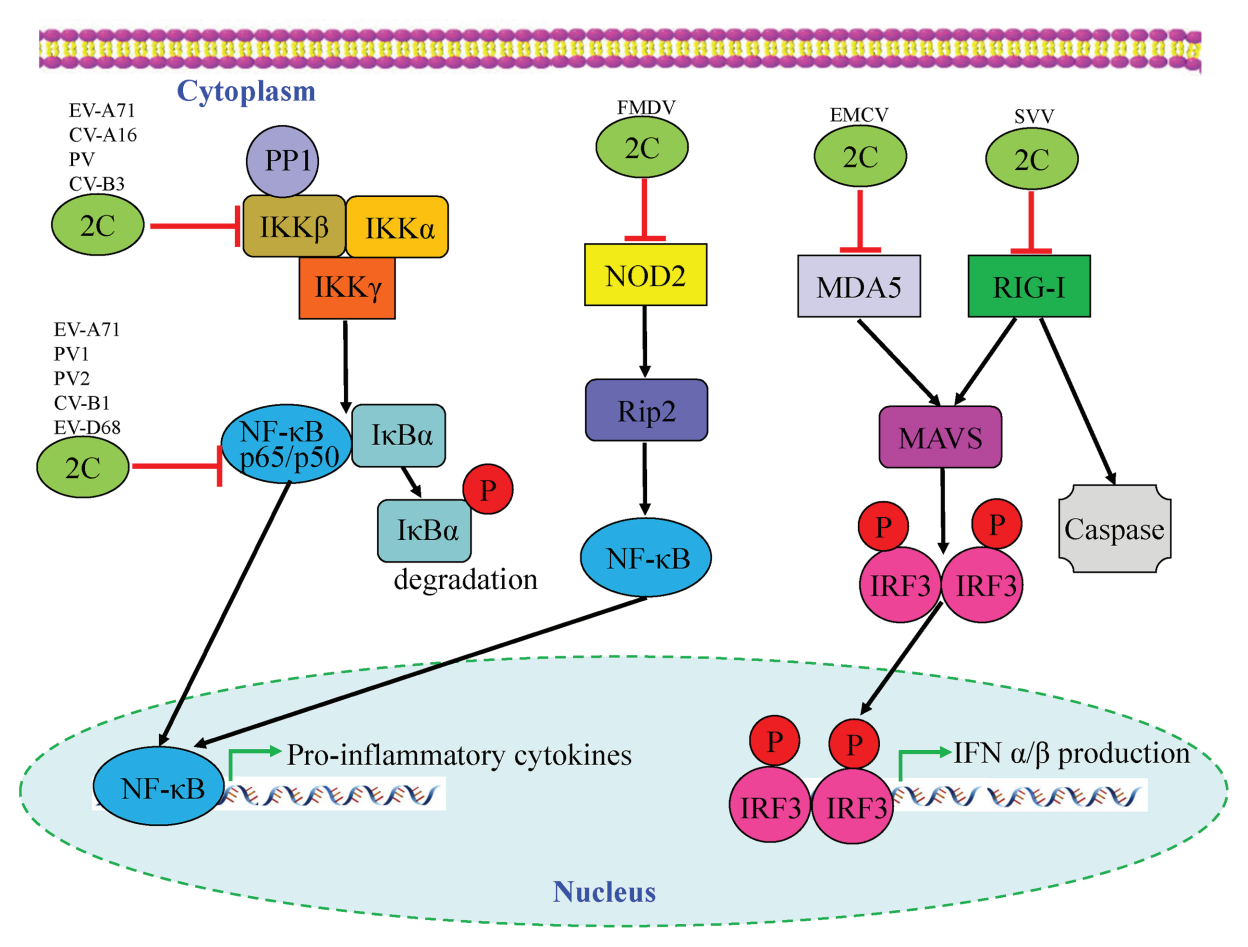
Interactions between picornavirus 2C protein and the NF-κB and retinoic acid-inducible gene I (RIG-I)-like receptor (RLR) pathways. Enterovirus 2C proteins are mainly involved in downregulation of pro-inflammatory cytokines by targeting the NF-κB pathway, while foot-and-mouth disease virus (FMDV) 2C regulates the corresponding pathway by suppressing the expression of NOD-2. Two picornavirus 2C proteins, including encephalomyocarditis virus (EMCV) and seneca valley virus (SVV), are shown to target the MDA5 and RIG-I of the RLR pathway which may cause downstream mediators to counteract antiviral innate immunity.

Many viruses have evolved mechanisms to regulate the NF-κB pathway for viral replication and cell survival to evade host immune responses. As shown in Figure 4, Zheng et al. (2011) found that the phosphorylation of IKKβ is inhibited by EV-A71 2C protein, thereby blocking TNF-α-mediated NF-κB activation. Specifically, 2C can directly bind to the KD domain of IKKβ through the 1–125 aa of the N-terminal to inhibit IKKβ phosphorylation (Zheng et al., 2011). Further research by Zheng et al. indicated that EV-A71 2C interacts with protein phosphatase 1 (PP1), recruits PP1 to IKKβ, and finally forms a 2C-PP1-IKKβ complex to inhibit IKKβ phosphorylation and the subsequent NF-κB signaling pathway. CV-A16 2C, CV-B3 2C, and PV 2C also possess the ability to suppress IKKβ phosphorylation in the same way as EV-A71 2C (Li et al., 2016b). The 2C protein is associated with both virus replication and innate immune evasion. Du et al. (2015) reported two different pathways by which NF-κB activation is suppressed by EV-A71 2C protein. One was RelA (p65)/p50, the predominant form of NF-κB; its dimerization is inhibited by the 105–125 and 126–203 aa of EV-A71 2C, competing to interact with the IPT domain of p65, thus releasing the association between p65 and p50. Another mechanism is the suppression of NF-κB activation by the 1–104 and 105–121 aa of 2C through association with IKKβ (Figure 4; Table 2).

**Table 2:** Mechanism in suppressing the production of IFNs, inhibiting virus replication, and inducing autophagy by picornavirus 2C proteins.

Protein	Genus	Species	Functions	Mechanism	References
2C	*Enterovirus*	EV-A71; EV-A71, CV-A16, PV, CV-B3; EV-A71, PV1, PV2, CV-B1, EV-D68	Inhibits NF-κB signaling pathway	By interaction with IKKβ; by interaction with IKKβ and PP1; suppressing p65/p50 dimerization by competing p65 IPT domain and by targeting Rel(A) and IKKβ	Zheng et al., 2011; Du et al., 2015; Li et al., 2016b
2C	*Aphthovirus*	FMDV	Antagonizes NOD2-mediated IFN-β and NF-κB signaling pathways	By interaction between NOD2 and the ATPase domain of 2C	Liu et al., 2019
2C	*Senecavirus*	SVV	Inhibit the production of IFN-β	By inducing the degradation of RIG-I	Wen et al., 2019
2C	*Cardiovirus*	EMCV	Suppress IFN-β signaling pathway	By interaction with MDA5	Li et al., 2019a
2C	*Enterovirus*	EV-A71	Viral replication	By interaction with COPI	Wang et al., 2012b
2C	*Enterovirus*	EV-A71, CV-A16, PV	Viral replication	By interaction with RTN3	Tang et al., 2007
2C	*Enterovirus*	EV-A71, PV1, PV2, CV-B1, EV-D68	Viral replication	By interaction with TRIM4, exportin2, and ARFGAP1	Li et al., 2019b
2C	*Aphthovirus*	FMDV	Viral replication	By interaction with Beclin1	Gladue et al., 2012
2C	*Enterovirus*	PV	Negatively regulate 3C protease activity	By interaction with 3C	Banerjee et al., 2004
2BC	*Enterovirus*	EV-A71	Autophagy	By interaction with SNARE proteins	Lai et al., 2017
2C	*Enterovirus*	EV-A71	Autophagy	By colocalize with LC3 and MPR	Lee et al., 2014
2C	*Enterovirus*	EV-A71, CV-A16, CV-A6, EV-D68	Autophagy	By induce A3G degradation	Li et al., 2018c
2C	*Enterovirus*	CV-A6	Autophagy	Unclear	Wang et al., 2018
2C	*Enterovirus*	CV-A16	Autophagy	By increase IRGM promoter activation and enhance IRGM protein expression	Shi et al., 2015
2C	*Cardiovirus*	EMCV	Autophagy	Activate ER stress molecules and regulate proteins expression of UPR pathway	Hou et al., 2014

To date, there have been relatively few studies on enterovirus 2C proteins in the RLR and NLR pathways. However, the non-structural 2C proteins of other RNA viruses in the *Picornaviridae* family have been reported to be associated with the RLR pathway. As shown in Figure 4, the encephalomyocarditis virus (EMCV, *Cardiovirus* genus) 2C protein was reported to interact with MDA5 to suppress the induction of IFN-β expression (Li et al., 2019a). The suppression of IFN-β promoter activity and the ability to interact with MDA5 were reduced or lost by the V26 mutation of the EMCV 2C protein. In addition, V26A and K25-3A mutants of EMCV 2C abolished the effect of reducing the phosphorylation of IRF3. Wen et al. (2019) revealed that the 2C and 3C proteins of the seneca valley virus (SVV, *Senecavirus* genus) could weaken the host innate immune system by the degradation of RIG-I through the caspase signaling pathway. Recently, NLRs have been demonstrated to play vital roles in the host immune response during viral infection (Lupfer and Kanneganti, 2013). Liu et al. demonstrated that as well as 2B and 3C, the foot-and-mouth disease virus (FMDV, *Aphthovirus* genus) 2C protein can also reduce the expression of the protein levels of NOD2, a novel cytoplasmic viral pattern recognition receptor identified in 2009 (Liu et al., 2019). However, the mechanism of reduction of NOD2 by FMDV 2C does not involve proteasomes, lysosomes, caspases, cellular apoptosis, or cleavage of eIF4G. The 116–260 truncation of FMDV 2C was demonstrated to play a vital role in interactions with NOD2, but a reduction of NOD2 expression was not induced by truncated 2C mutants (Figure 4; Table 2). In conclusion, host cells have developed multiple strategies against viral infections; however, viruses have evolved many antagonistic mechanisms to escape the host innate immune response. These studies have led to the identification of the critical roles of 2C proteins as immunomodulatory property regulators. Further studies will not only investigate the host immune response activation suppression by 2C proteins of the main circulating enteroviruses but also provide a general understanding of picornavirus 2C proteins, as vital mechanisms that are likely to be conserved in most of the picornaviruses.

## Regulation of Host Cell Autophagy

Autophagy is a conserved intracellular process which acts to remove unnecessary or dysfunctional cytoplasmic proteins and damaged or obsolete organelles by delivering them to lysosomes for degradation and recycling (Klionsky, 2005; Esclatine et al., 2009). Our previous study was the first to report that viral protein 2C of CV-A6 contributed to the pathogenicity of CV-A6 by inducing cell death through the autophagy pathway (Wang et al., 2018), but the mechanism underlying this phenomenon needs to be further elucidated (Table 2).

In 2009, Huang et al. (2009) reported that EV-A71 infection can induce autophagy and increase viral replication both *in vitro* and *in vivo*. EV-A71 2C was identified to colocalize with microtubule-associated protein 1 light chain 3 (LC3) and mannose-6-phosphate receptor (MPR), indicating the potential for amphisome formation and autophagy induction (Lee et al., 2014). Recently, Li et al. (2018c) reported that the EV-A71 2C protein could overcome host restriction factor APOBEC3G (A3G) suppression through the autophagy-lysosome pathway, whose functions are conserved among EV-D68, CV-A6, and CV-A16. The autophagosome-lysosome fusion process is regulated by a family of *N*-ethylmaleimide-sensitive factor attachment receptor (SNARE) proteins (Wang et al., 2016). SNARE fusion bundles require four α helices to function, including Qa, Qb, Qc, and R (Rizo, 2003). Syntaxin-17 (STX17) is the Qa SNARE on the completed autophagosome, which can coordinate its fusion with other vesicles (Itakura et al., 2012). Synaptosome-associated protein 29 (SNAP29) is a cytosolic Qbc SNARE that can donate its helices to the formation of a fusion bundle by interaction with STX17 (Morelli et al., 2014). The non-structural protein 2BC of EV-A71, which is a precursor protein of 2B and 2C, has been reported to trigger the formation of autolysosomes, which facilitate virus replication by interacting with both SNARE proteins STX17 and SNAP29 (Table 2; Lai et al., 2017). Recently, Shi et al. (2015) found that the 2C protein of CV-A16 is sufficient to induce incomplete autophagy. The immunity-related GTPase family M (IRGM) promoter activity and the protein expression levels are enhanced by the expression of CV-A16 2C, and subsequently induce autophagy. Non-structural protein 2C of EMCV is involved in triggering autophagy induced by EMCV infection in BHK-21 cells (Hou et al., 2014). Autophagy is induced by EMCV 2C through activation of the ER stress pathway by regulating the expression of PERK and ATF6α, which are involved in the UPR pathway. The 2C protein of FMDV is co-localized with LC3, an autophagosome marker, in FMDV-infected cells, indicating the potential for induction of autophagy by FMDV 2C (O’Donnell et al., 2011). The 2C protein of FMDV binds to Beclin1, a central regulator of the autophagy pathway, thereby repressing the fusion of lysosomes to autophagosomes and subsequent viral survival (Table 2; Gladue et al., 2012). The relationship between the viral 2C protein and host cell autophagy-related proteins needs to be further studied to better evaluate the role of the 2C protein in virus infection.

## Effect of Anti-Viral Drugs on the 2C Protein

Within the *Enterovirus* genus, there are two effective vaccines for two human pathogens, PV and EV-A71. However, the current total of human enteroviruses exceeds several hundred serotypes, and the development of vaccines against all enteroviruses is unlikely to be achieved. Severe, life-threatening illness can occur, especially in young children, due to enterovirus infection. Thus, there is an urgent need for the development of new anti-viral drugs against different types of enteroviruses (Ulferts et al., 2016).

As 2C is a highly conserved viral non-structural protein, possesses ATPase activity and is functionally indispensable, it is a promising target for drug development involving broad-spectrum enterovirus inhibitors (Bauer et al., 2017). To date, several anti-viral inhibitors which target the 2C protein have been identified, including guanidine hydrochloride (GuHCl; Pfister and Wimmer, 1999; Sadeghipour et al., 2012), HBB (Hadaschik et al., 1999), MRL-1237 (Shimizu et al., 2000), and TBZE-029 (De Palma et al., 2008a). In addition, several other compounds, such as metrifudil (Arita et al., 2008), *N*^6^-benzyladenosine (Arita et al., 2008), quinoline analogues (Musharrafieh et al., 2019), dibucaine derivatives (Tang et al., 2020), fluoxetine analogues (Manganaro et al., 2020), R523062 (Ma et al., 2020), and viperin (Wei et al., 2018), which also target the enterovirus 2C protein, are summarized in Table 3.

**Table 3:** Literature reported anti-viral drugs targeting enterovirus 2C proteins.

Drugs	Species	Drug-resistant mutations	References
GuHCl	PV; PV, CV-B3	I142V, N179G, M187L; A143G, S225T, I227M, A233T/S; A224V, I227V, A229V	Pincus et al., 1986; Tolskaya et al., 1994; De Palma et al., 2008a
Fluoxetine	CV-B3; CV-B3, EV-D68	C179F, F190L; A224V, I227V, A229V	Ulferts et al., 2013; Bauer et al., 2019
Fluoxetine analogues 2b	CV-B3, EV-D68	A224V, I227V, A229V, C179F	Manganaro et al., 2020
TBZE-029	CV-B3	A224V, I227V, A229V	De Palma et al., 2008a
HBB	CV-B3	A224V, I227V, A229V	De Palma et al., 2008a
MRL-1237	PV; CV-B3	I120V, F164Y; A224V, I227V, A229V	Shimizu et al., 2000; De Palma et al., 2008a
Pirlindole	CV-B3	A224V, I227V, A229V	Ulferts et al., 2016
Dibucaine	CV-B3	A224V, I227V, A229V	Ulferts et al., 2016
Dibucaine derivatives 6i	EV-A71	ND	Tang et al., 2020
Zuclopenthixol	CV-B3	A224V, I227V, A229V	Ulferts et al., 2016
Metrifudil	EV-A71	E325G	Arita et al., 2008
*N*^6^-benzyladenosine	EV-A71	H118Y, I324M	Arita et al., 2008
Quinoline analogues 10a, 12a, 12c	EV-D68	ND	Musharrafieh et al., 2019
R523062	EV-D68	I227L	Ma et al., 2020
Viperin	EV-A71	ND	Wei et al., 2018

ND, not determined.

### Guanidine Hydrochloride

Of these drugs, GuHCl is the most extensively studied (Rightsel et al., 1961; Loddo et al., 1962). GuHCl is an FDA-approved small compound drug that has been used for the treatment of the autoimmune disorder disease Lambert-Eaton myasthenic syndrome (Lambert, 1966). GuHCl can inhibit the replication of several picornaviruses, including PV, coxsackieviruses, echoviruses, and FMDV, but not HAV (De Palma et al., 2008b). Early *in vitro* studies have shown that the initiation of viral RNA synthesis is inhibited by GuHCl (Tershak, 1982). It has been shown that GuHCl suppresses 2C function, which is required for the initiation of negative- but not positive-strand RNA synthesis, and RNA chain elongation of PV (Barton and Flanegan, 1997). Several studies into the resistance and/or dependence of PV and FMDV to GuHCl attributed this resistance to the 2C protein (Saunders et al., 1985; Pincus et al., 1986, 1987; Baltera and Tershak, 1989; Tolskaya et al., 1994). The resistance of PV to GuHCl was attributed primarily to mutations at positions 179 and 187 of 2C (Pincus et al., 1986; Tolskaya et al., 1994). Pfister et al. (2000) found that ATP hydrolysis activity can be inhibited by GuHCl at millimolar concentrations, and the resistance and dependence of GuHCl were also attributed to 2C (Pfister and Wimmer, 1999).

It has long been considered that the helicase of viruses is a potential target for anti-viral drug development due to its importance in viral RNA replication (Kwong et al., 2005). Previous studies have demonstrated that the ATPase activity of PV 2C could be inhibited by GuHCl (Pfister and Wimmer, 1999). Similarly, serial studies have reported that GuHCl can inhibit the NTPase and helicase activities of several helicases, including the 2C protein of EV-A71, NS3 protein of human norovirus, and VP35 protein of Ebola virus (EBOV), as well as inhibit the RNA replication of enterovirus, norovirus, and EBOV (Xia et al., 2015; Li et al., 2018b; Shu et al., 2019). Guanidines are universally present in the environment and may bind to the surface of viral RNA remodeling proteins to change their conformations, electrostatic states, and protein-protein or protein-RNA interactions, ultimately inhibiting the corresponding RNA remodeling activities (Shu et al., 2019). Because toxicity concerns of GuHCl may prevent its clinical use, recent drug development for guanidine derivatives have identified several potential anti-viral drugs against HCV, human immunodeficiency virus (HIV), and flaviviruses (Frick, 2007; Saczewski and Balewski, 2009, 2013; Lindquist and Stangel, 2011).

### Drug Repurposing

Recently, drug repurposing has become of increasing interest, as the pharmacological and toxicological information related to many drugs are already available, and when a repurposed drug is used at a similar dosage as it was in its original application, it may directly enter phase II clinical trials, thereby saving development cost and time. To date, fluoxetine, pirlindole, dibucaine, and zuclopenthixol, all FDA-approved drugs, have been identified using drug repurposing screens, as targeting 2C proteins and inhibiting the replication of enterovirus species B and D members (Ulferts et al., 2013, 2016).

Fluoxetine, which is a selective serotonin reuptake inhibitor, selectively suppresses the replication of EV-B and EV-D, but not EV-A, EV-C, or rhinovirus A or B (RV-A or -B; Ulferts et al., 2013). TBZE-029, which is also a 2C targeting compound, could inhibit the growth of EV-B and EV-D, but not EV-A or EV-C (Ulferts et al., 2013). In 2019, Bauer et al. (2019) reported that the *S*-enantiomer of fluoxetine inhibits enterovirus replication by directly binding to the 2C protein of CV-B3. A substitution at positions A224, I227, and A229 of the 2C protein, which are located in the short stretch of amino acids 224AGSINA229 and the C-terminal of ATPase motif C, could confer resistance to fluoxetine (Ulferts et al., 2013). This 224AGSINA229 motif is conserved between EV-B (CV-B3) and EV-D (EV-D68) but not in other species of enteroviruses (Bauer et al., 2019). The differences mean that some of these mutations can confer resistance to fluoxetine, indicating the vital role of the substitution sensitivity of these viruses to this inhibitor (Ulferts et al., 2013). A mutation in the 224AGSINA229 loop not only confers resistance to fluoxetine but also to several other compounds, including TBZE-029, HBB, MRL-1237, and GuHCl (De Palma et al., 2008a; Ulferts et al., 2013). The authors built a homology model of CV-B3 2C based on the crystal structure of the published EV-A71 2C (Guan et al., 2017) to investigate the mechanism by which fluoxetine binds to CV-B3 2C (Bauer et al., 2019). Unfortunately, the 2C mutations at the bottom (positions I227V, C179F, and F190L) and on the borders (positions V187M and D245N) of the predicted pocket against (*S*)-fluoxetine binding activities were completely opposite, so the entrance sites that fluoxetine used to the hydrophobic cavity of 2C could not be confirmed (Bauer et al., 2019).

Recently, Ulferts et al. (2016) identified pirlindole as a novel inhibitor by screening FDA-approved drugs against CV-B3. They found that EV-B and EV-D could both be suppressed by pirlindole and dibucaine, and dibucaine could also inhibit EV-A, but none of these compounds could inhibit EV-C or RVs. All of these compounds exert an inhibitory effect by acting at the stage of genome replication. Further studies revealed that A224V, I227V, and A229V mutations of 2C provide resistance to pirlindole, dibucaine, and zuclopenthixol, a finding which was consistent with the effects of GuHCl and fluoxetine in anti-enteroviral treatment. However, formoterol, which could inhibit all tested enteroviruses and RVs, does not target 2C, and its mechanism of action needs to be further elucidated (Ulferts et al., 2016).

### Outlook

Collectively, future anti-viral drug development against enterovirus infection will focus on the RNA remodeling activity of 2C protein, to modify and screen various guanidine derivatives with better inhibitory effects and lower toxicity. Fluoxetine, which was once used to treat major depression and anxiety disorders, has more recently been used as an effective inhibitor for immunocompromised children with chronic enterovirus encephalitis (Gofshteyn et al., 2016), demonstrating its potential for clinical use as an enterovirus 2C inhibitor. So far, these 2C inhibitors have not been approved for clinical application in the treatment of enteroviral infections. Hence, we need more crystallographic data on the types of enterovirus 2C proteins, to clarify the underlying mechanisms of the inhibitors’ efficacy, drug resistance, and exact binding style, and to facilitate the rational design of fluoxetine-derived and developing novel broad-spectrum enterovirus drugs.

## Conclusion and Future Perspectives

Enteroviruses are the main causes of HFMD, with EV-A71, CV-A16, CV-A6, and CV-A10 being the main circulating pathogens of HFMD, especially in the Asia-Pacific region. The non-structural 2C protein is the most highly conserved of the enteroviral proteins but is still poorly understood. In this review, we focused on the current understanding of the structure and multi-functionality of the 2C proteins of enteroviruses, and the different roles of 2C proteins and host innate immunity. In the past two decades, PV 2C has been the most-studied non-structural 2C protein. Previous studies have demonstrated that the PV 2C protein possesses multiple activities in the viral life cycle (Table 1). The 2C proteins of EV-A71 and CV-A16 were first revealed to have ATP-dependent RNA helicase and ATP-independent chaperoning activities, which are critical for viral RNA replication (Xia et al., 2015). In the present review, we summarized and discussed the current understanding of the relationships between structure and function of the enterovirus 2C proteins, especially the key mutations and motifs involved in viral infection and replication (Figure 2). Future studies will focus on the determination of additional crystal structures of enterovirus 2C proteins, data which will help us to elucidate the detailed mechanism of 2C protein involvement in virus replication and packaging.

In this review, we summarized novel host factors such as RTN3, COPI, TRIM4, exportin2, and ARFGAP1, which have been demonstrated to interact with enterovirus 2C proteins to contribute to viral replication (Figure 3; Table 2). Further studies will identify additional host factors that interact with 2C, help us to better understand enterovirus biology, and provide new targets for the development of anti-viral therapy. It is important to investigate the conservation of these host factors among other enteroviruses and even picornaviruses.

The innate immune system is the first line of defense against viral infections, and thus triggers adaptive immunity, which plays vital roles in the fight against viral infections, especially the induction of type I IFN response. Several studies have reported that picornavirus 2C proteins participate in host innate immunity by associating with the NF-κB, MDA5, RIG-I, NOD2, and IFN signaling pathways (Figure 4; Table 2), which provide the mechanisms for evasion of the innate immune response during viral infection. Further studies will focus on the detailed mechanisms by which 2C proteins target and regulate the NF-κB pathway and the three major classes of PRRs, which will ultimately lead to clarification of the interplay between PRRs and the innate immune system.

Currently, although there has been successful use of a vaccine against EV-A71, a novel vaccine against all enteroviruses or multiple circulating causative pathogens is unavailable. Thus, there is an urgent need for novel anti-viral drugs, especially broad-spectrum anti-viral drugs, to treat multiple enterovirus infections. The broad-spectrum anti-enteroviral fluoxetine is considered as the most promising 2C inhibitor, but detailed mode-of-action studies are still missing, as the crystal structure of the 2C protein, which is derived from fluoxetine-sensitive enteroviruses, has still not been solved (Table 3). Recently, the crystal structures of EV-A71 2C and PV 2C have been resolved (Guan et al., 2017, 2018). However, the crystal structures of other enterovirus 2C proteins, especially the main circulating pathogens such as CV-A16, CV-A6, and CV-A10, have not been determined. Future studies will focus on the determination of the crystal structure of different types of enterovirus 2C proteins and screening of broader anti-viral drugs, which will help us to elucidate the pathogenesis of enteroviral infections, and facilitate the development and application of 2C inhibitors for clinical treatment for enteroviral infections.

## Author Contributions

S-HW is the first author of this article and wrote the manuscript. KW and KZ had made contributions to this article. S-CH and JD had discussed and revised this article. All authors contributed to the article and approved the submitted version.

### Conflict of Interest

The authors declare that the research was conducted in the absence of any commercial or financial relationships that could be construed as a potential conflict of interest.
